# Reading and spelling skills of prematurely born children in light of the underlying cognitive factors

**DOI:** 10.1007/s10339-020-01001-6

**Published:** 2020-10-27

**Authors:** Rózsa Gráf, Magda Kalmár, Andrea Harnos, Gábor Boross, Anett Nagy

**Affiliations:** 1 Institute of Teacher Trainig, Section of Special Education, University of Miskolc, Miskolc, Hungary; 2 Department of Neonatology and Neonatal Intensive Care Unit, Péterfy Sándor Hospital, Budapest, Budapest, Hungary; 3 Institute of Psychology, Eötvös Loránd University of Budapest, Budapest, Hungary; 4 Faculty of Special Education, Eötvös Loránd University of Budapest, Budapest, Hungary; 5 Department of Biomathematics and Informatics, University of Veterinary Medicine, Budapest, Budapest, Hungary

**Keywords:** Low birthweight, Learning difficulty, Dyslexia, 3DM, CART

## Abstract

Prematurity is a serious risk factor for learning difficulties. Within the academic skills reading has the greatest impact on the prospects of the students; therefore, studying the reading skills in the risk populations is very important. The aim of our study was to investigate reading and spelling skills of prematurely born children. Our target group consisted of 8–11-year-old children (*n* = 23) who were born preterm with very low birthweights (VLBW). For comparison 57 full-term children (27 good readers and 30 dyslexics) were included in the study sample. To assess the reading and spelling abilities the Hungarian version of the 3DM (Dyslexia Differential Diagnosis) was used. Cognitive abilities were tested using the Hungarian adaptation of the WISC-IV and the Rey Complex Figure Test. The data were analyzed with a novel statistical approach using the R program. In the cognitive measures the mean performances of all three groups fell within the normal range. In the WISC-IV Full-scale IQ as well as in some other cognitive measures the good readers significantly outperformed both the dyslexics and the preterms. The findings of the study did not confirm our expectation that VLBW prematurity should lead to developmental disadvantages in the acquisition of reading and spelling skills since in the reading and spelling performances of the good readers and the preterms did not differ, while both the good readers and the preterms scored higher than the dyslexics. The results suggest that the cognitive assets of the preterm children contributing to their reading and spelling performances were their good spatial–visual memory, working memory, and processing speed. The identification of the cognitive mechanisms underlying reading and spelling abilities is of crucial importance for designing intervention for children with deficits in these academic skills.

## Introduction

### Background

Premature birth (before the 37 week of gestation) is the most common perinatal risk; therefore, the heightened research interest in the development of preterm children is not surprising. In spite of the ever-growing bulk of research evidence, the picture is far from being clear. As far as the long-term outcome is concerned, the majority of the studies reported IQ’s of moderate-risk preterm children significantly lower as compared to those of full-term comparison groups (Aylward 2002; Hadders-Algra 2005; Kalmár 1996; Rose et al. 2005), mostly falling into the lower third of the average zone (Breslau and Chilcoat 1996; Breslau et al. 2001; Rose et al. 2011). Several researchers found cognitive deficits in various domains associated with structural disorders of the brain (Inder et al. 2005; Bradley et al. 2000; Skranes et al. 2012). School-age preterm children even with normal IQs and without neurological impairment often display cognitive dysfunctions mainly related to visual processing and executive functions (Larsson et al. 2005).

Prematurity is a serious risk factor for learning difficulties (Aarnoudse-Moens et al. 2006; Breslau et al. 2001; Saigal et al. 1991). Within the academic skills reading is likely to have the greatest impact on the prospects of the students. Reading deficits hamper school performance across various domains and, hence, often lead to the need of special education as well as to failures in the adult life (Kovachy et al. 2014; Samuelsson et al. 2006). Studying the reading skills in the risk populations is therefore a research issue of special importance.

Reading has two fundamental components: decoding (single-word reading) and reading comprehension, i.e., to derive meanings from and form interpretations of written words and sentences. To acquire reading, first one should learn the code for representing speech as a series of visual symbols. It is a process of matching visual symbols (in many languages letters) to units of sound. Decoding stems from primary linguistic skills such as phonological awareness and alphabet knowledge. Efficient reading also requires the recognition of the orthographic patterns of whole words not only accurately but also fluently, that is, automatically. The acquisition of this skill which is a basis of comprehension is a further step in learning to read. Comprehension is more complex as it requires the integration of these linguistic skills with higher-order cognitive processes, e.g., working memory (Kovachy et al. 2014; Shaywitz and Shaywitz 2008; Ziegler and Goswami 2005).

A recent meta-analysis by Kovachy et al. (2014) covering studies which compared the reading abilities in school-age children who were born preterm to those in full-term comparison groups demonstrated that the performances of the preterm children were significantly worse on both major components of reading, i.e., on decoding and reading comprehension. The preterm samples of the studies included in the meta-analysis were rather heterogeneous. The upper limit of gestational age was 32 weeks, and the mean birthweights varied between 740 and 1256 g. Gestational age was significantly associated with the reading performances in the preterms: Lower gestational ages increased the disadvantages. However, the group differences remained the same after the exclusion of children with major disabilities or intellectual impairments as well as after controlling for the inequalities of the socio-economic status. In the above meta-analysis the effect of birth weight was not checked, although it is generally acknowledged to contribute to the degree of risk in prematurely born children. A considerable proportion of research limits their samples to very low birthweight (< 1500 g, VLBW) preterms. Two research teams (Samuelsson et al. 2006; Takeuchi et al. 2016) which investigated the reading abilities in elementary school-age VLBW children with very different native languages reported remarkably similar findings: Roughly one-third of both the Swedish and the Japanese VLBW children with normal intelligence had reading difficulties. The 9-year-old Swedish VLBW children were more disadvantaged in the orthographic (spelling-based) reading skills than in the phonological (sound-based) skills (Samuelsson et al. 2006). Language functions such as vocabulary and receptive language were found relatively intact in VLBW children (Aylward 2002; Ment et al. 2003) which suggest that the mechanism of reading difficulty in VLBW preterms differs from that in full-term children. A potential complication of VLBW preterm birth is a periventricular white matter injury which may compromise the visual cognition pathway (Downie et al. 2003). Problems in visual cognition, in addition to phonological weakness, might play an important role in the reading difficulties in VLBW children (Takeuchi et al. 2016).

However, the literature on the reading abilities of preterm children is not consistent. A number of studies did not find the reading performances of the preterm groups significantly different from those of the full-term comparison groups (Kesler et al. 2004; Lee et al. 2017; Takeuchi et al. 2016; Taylor et al. 2011). It is also notable that the reading deficits in the Swedish VLBW children followed up by Samuelsson et al. (2006) did not persist into adolescence. By 15 years of age the VLBW subjects free of identified brain insults caught up with their non-risk counterparts on all reading measures, and the comparison between the total VLBW sample and the full-term group remained significant on one measure only.

The specific characteristics of the different languages are likely to influence the learning processes in the acquisition of reading (Ziegler and Goswami 2005); therefore, the research in foreign languages cannot provide full guidelines for the measurement of the reading skills and the interpretation of the potential faults and deficits.

The aim of our study was to investigate certain reading and spelling skills of school-age prematurely born Hungarian children. Based on the majority of research evidence available in the literature our hypothesis was that the reading and spelling performances of the preterm children will be poorer than those of the same-age non-risk, problem-free children. According to our knowledge no research has so far compared the development of reading and spelling skills in preterm children and that of children diagnosed with dyslexia. We expected that the reading deficits in the preterm children will not be as severe as those in the dyslexics. We were also interested in the cognitive mechanisms underlying the reading and spelling performances and whether those mechanisms differ depending on the children’s birth status (full-term—preterm) and reading disability diagnosis.

## Methods

### Subjects

The target group consisted of school-age children (*n* = 23) who were born preterm with very low birthweights (990–1350 g, mean: 1211.7 g), at 28–33 weeks (mean: 30 weeks) of gestation and cared for in the Neonatology Ward of the Péterfy Sándor Hospital in Budapest. They had no neurological impairments and were considered at moderate risk. The preterm children were compared to two groups of full-term children. Thirty of the full-term children were dyslexics, and 27 of them were good readers. Children with seizures, attention disorders (scoring > 70 on the attention problem subscale of the CBCL, Achenbach and Rescorla 2001), uncorrected visual problems, specific language impairment, and language perception disorders were not included in the study. The groups were matched on age. The age means and ranges in the three groups were the following: preterms mean 9.56 years (7.6–11.1); full-term dyslexics mean 10.21 years (7.8–12.8); full-term good readers mean 9.67 years (7.1–10.9).

The children attended Hungarian-language schools (grade 2–4) and were not absent from school more than 3 months. At least one of the parents used Hungarian in communicating with the child since his/her birth. None of the children came from socially disadvantaged backgrounds, and all the mothers completed at least 8 years of general school. All of the children scored > 85 both on the verbal comprehension index and the perceptual reasoning index of the Hungarian version of the WISC-IV. The aim of the study did not require a more precise matching of the groups on IQ as it was a question to what extent and which way the various aspects of intelligence explained the individual variances in reading and spelling performances.

### Instruments

The Hungarian version of the 3DM^1^ (Dyslexia Differential Diagnosis, Maastricht; Blomert and Vaessen 2009; 3DM-H: copyright Csépe et al. 2009; Ziegler et al. 2010) was used to test reading and spelling abilities. The reading test consists of common and rare real words as well as pseudo-words appearing on a computer screen, the difficulty of which both in length and orthography are gradually increasing. In the spelling test incomplete words appear on the screen which are to be completed by the subject, using letters offered by the computer. Dyslexia was established using the reading test of the 3DM-H. The “good readers” and the “dyslexics” were identified on the basis of *z*-scores of accuracy and fluency in reading the presented words and pseudo-words (good readers: *T* value 48–60; dyslexics: *T* value below 32).

Cognitive abilities were tested using the Hungarian adaptation of the WISC-IV and the Rey Complex Figure Test (RCFT) (Ogino et al. 2008; Ohtuska 2008).

## Measures

*WISC-IV* Full Scale IQ (FSIQ), Verbal Comprehension Index (VCI), Perceptual Reasoning Index (PeRI), Working Memory Index (WMI), Processing Speed Index (PrSI).

*RCFT* Copy Time (Rey CT), Copy Score (Rey CS), Memory Time (Rey MT), Memory Score (Rey MS),

*3DM* Reading Accuracy (RA), Reading Fluency (RF), Spelling Accuracy (SA), Spelling Speed (SP).

### Data analysis

The data analysis was performed using the 3.4.0 version of the R statistical program (R Core Team 2017).

The performance scores were analyzed by general linear mixed models (GLMM), using the “nlme” package (Pinheiro et al. 2015).

The group was the fix factor in the models, and the school was the random factor to take the similarities within the schools into account. In case of significant results (*p* < 0.05), the estimated scores of the groups were compared by linear contrasts using Tukey adjustments of *p* values and 95% confidence limits (Tukey 1949).

The cognitive background of the reading and spelling abilities was tested applying Random Forests and Classification and Regression Trees (Hothorn et al. 2006a, b).

## Results

The scores of each child fell in the normal range in all cognitive measures (Table 1).

**Table 1:** Mean scores and (SDs) on the cognitive measures in the three groups

Group (*n*)	FSIQ	VCI	PeRI	WMI	PrSI	Rey CT	Rey CS	Rey MT	Rey MS
Preterms (23)	103.2 (12.4)	103.9 (8.74)	100.8 (13.6)	98.8 (13.1)	107.0 (14.7)	256.4 (90)	24.9 (5.74)	131.0 (59.1)	17.0 (6.36)
Dyslexics (27)	99.4 (8.98)	103.2 (10.4)	100.6 (8.15)	92.6 (13.0)	99.44 (8.98)	214.3 (102)	23.3 (6.27)	111.1 (54.4)	13.0 (6.68)
Good readers (30)	112.8 (11.5)	112.8 (13.71)	111.0 (11.3)	105.3 (8.81)	109.4 (12.1)	301.0 (96)	29.0 (4.42)	148.7 (48)	21.1 (5.89)

In the WISC-IV FSIQ the good readers significantly outperformed both the dyslexics (*p* < 0.001) and the preterms (*p* < 0.031), and the same was true for the VCI (*p* = 0.032, *p* = 0.047) and the PeRI (*p* < 0.002, *p* < 0.013). In the WMI and the PrSI the good readers had a significant advantage only over the dyslexics (*p* < 0.009, *p* < 0.013). In most of the WISC-IV measures there was no difference between the preterms and the dyslexics with the only exception of the PrSI in which case the preterms scored better (*p* < 0.05).

According to the GLMMs the groups had a significant effect on all the measures (Table 2).

**Table 2:** Results of the GLMM’s ANOVA tests for the cognitive measures

Measure (WISC-IV)	*F*	*p*
FSIQ	2899.3	0.001
VCI	4.281	0.018
PRI	8.0566	0.001
WMI	4.281	0.018
PrSI	8.8753	0.001

The good readers performed significantly better than the dyslexics on each Rey measures (CT: *p* = 0.004. CS: *p* = 0.001. MT: *p* = 0.027. MS: *p* < 0.001). The advantage of the good readers over the preterms was significant only in the CS (*p* < 0.024) and marginal in the MS (*p* = 0.054) in which the difference between the preterms and dyslexics was also marginally significant (*p* = 0.064).

### Reading and spelling

In each reading and spelling measure both the good readers and the preterms performed significantly better than the dyslexics (in all comparisons: *p* < 0.001). The scores of the good readers and the preterms did not differ significantly.

According to the GLMM the groups had significant effects on all the 3 measures.

In order to reveal the cognitive background of the reading and spelling abilities a 3-step analyses were performed. Considering the few measurement points and the relatively great number of independent variables, in order to select the meaningful explanatory variables first Random Forests (Hothorn et al. 2006a) were applied. RF is an ensemble of Classification and Regression Trees (see in the next paragraph). The trees are built using random subsets of the data and random subsets of variables chosen at branching point of the trees. The importance of the variables can be estimated by this method (Hothorn et al. 2006b; Strobl et al. 2008).

In the second step a Classification and Regression Tree (CART) was fitted using the previously selected important variables to refine the selection of the explanatory variables and discover the potential interaction; CARTs are built using a nonparametric regression approach. Both numerical and categorical variables can be used to build a tree. The general rule is to split the observations into two parts based on a predictor variable (root) and then to split the subset further based on another or the same variable on a recursive way (Hothorn et al. 2006b).

Finally, general linear mixed models (GLMM) were fitted with the previously selected explanatory variables to prove their significance. The linear mixed model made it feasible to take into consideration the dependency of the data (the children attending the same school cannot be considered independent).

### Reading accuracy

**Fig. 1:**
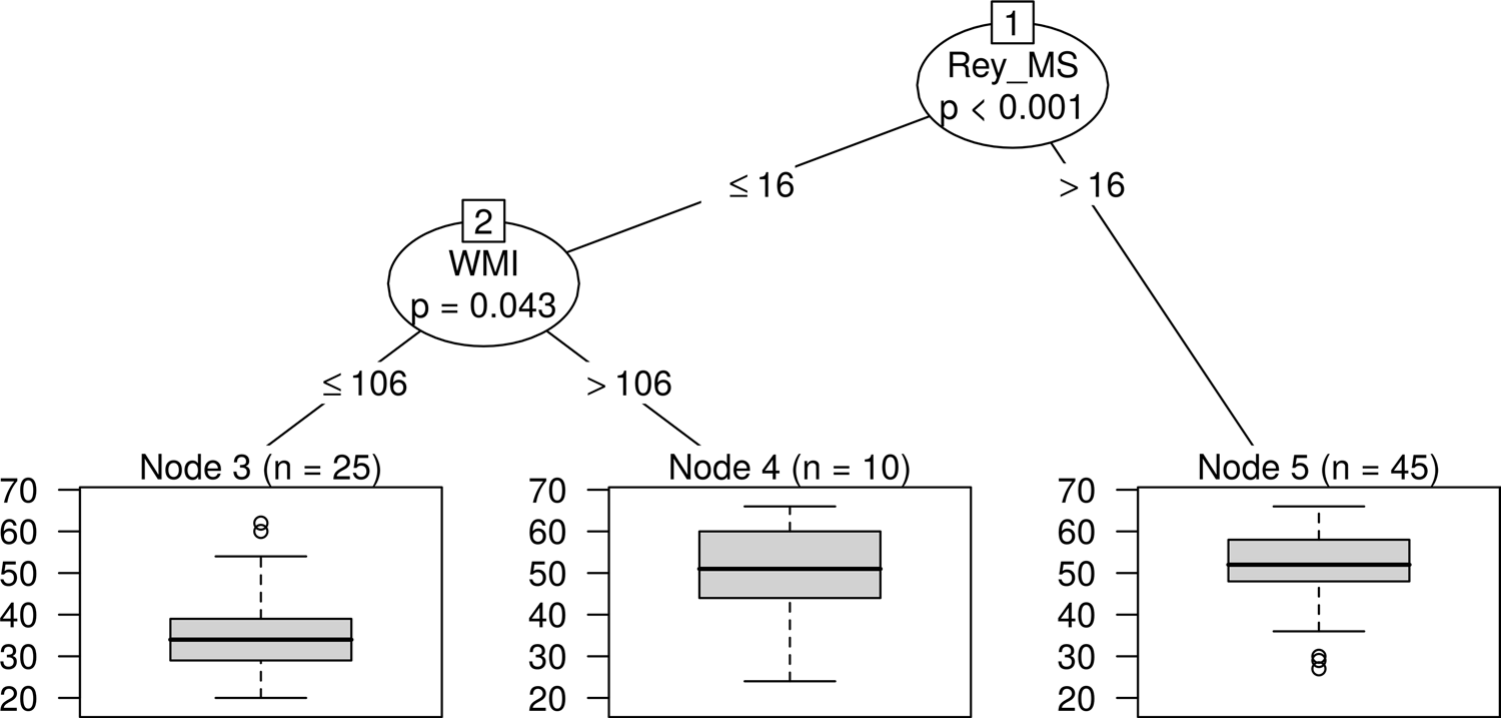
CART decision tree for reading accuracy

For reading accuracy the following explanatory variables were selected by the Random Forest: Rey MS, FSIQ, Rey CT, and WMI. On the ground of the intercorrelations, the CART eliminated the FSIQ and the Rey CT. The most powerful predictor was the recall accuracy of the Rey figures. The accurate readers had Rey MS > 16, but subjects with Rey MS ≤ 16 had chances to be accurate readers if scored > 106 on WMI. The WMI moderated the contribution of the Rey MS to reading accuracy (Fig. 1).

### Reading fluency

**Fig. 2:**
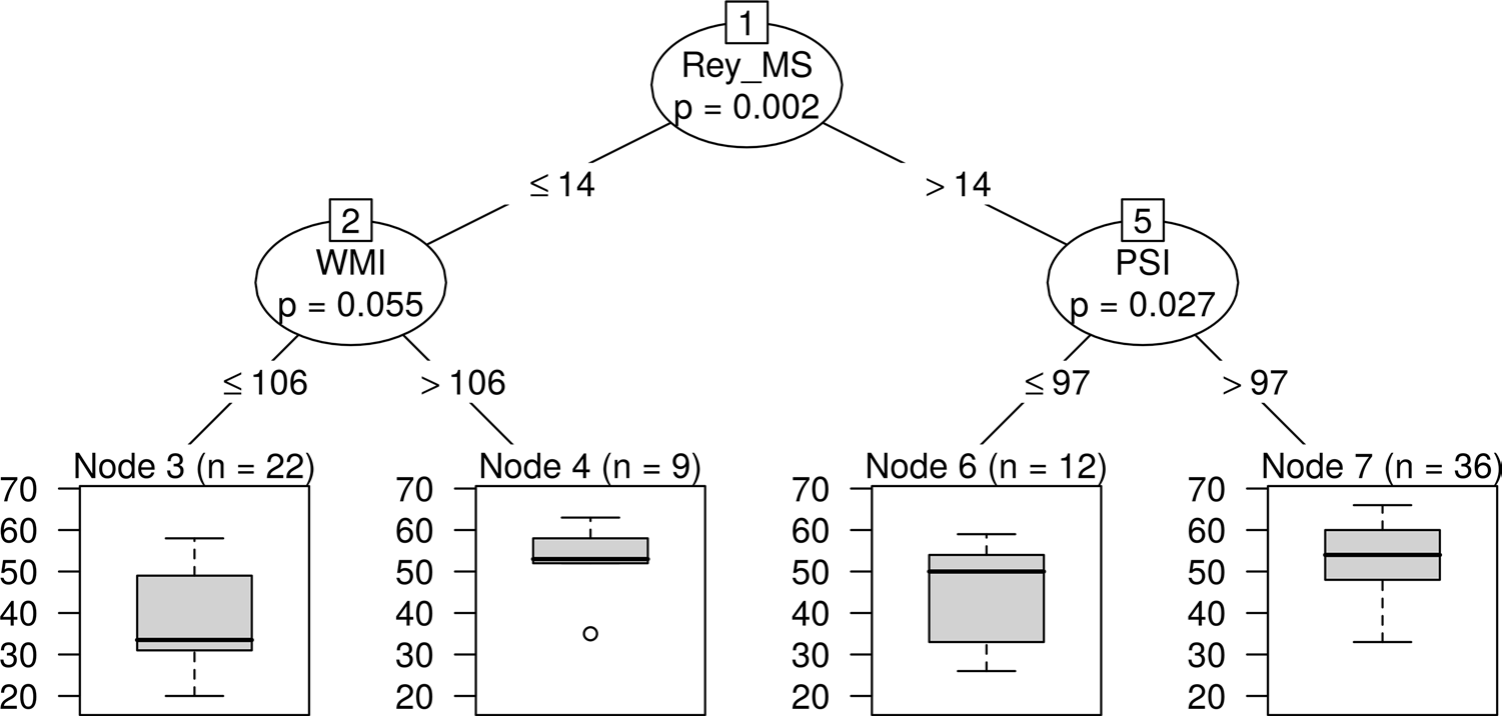
CART decision tree for reading fluency

For reading fluency the Rey MS, PrSI, and WMI came out as important variables at the first step which was corroborated by the CART technique. The most powerful predictor of fluent reading was the recall accuracy of the Rey figures (Rey MS). Working memory and processing speed had mediator roles: Children with lower Rey MS (≤ 14) but having relatively high WMI (> 106) and/or PrSI (> 97) could as well be fluent readers (Fig. 2).

### Spelling accuracy

**Fig. 3:**
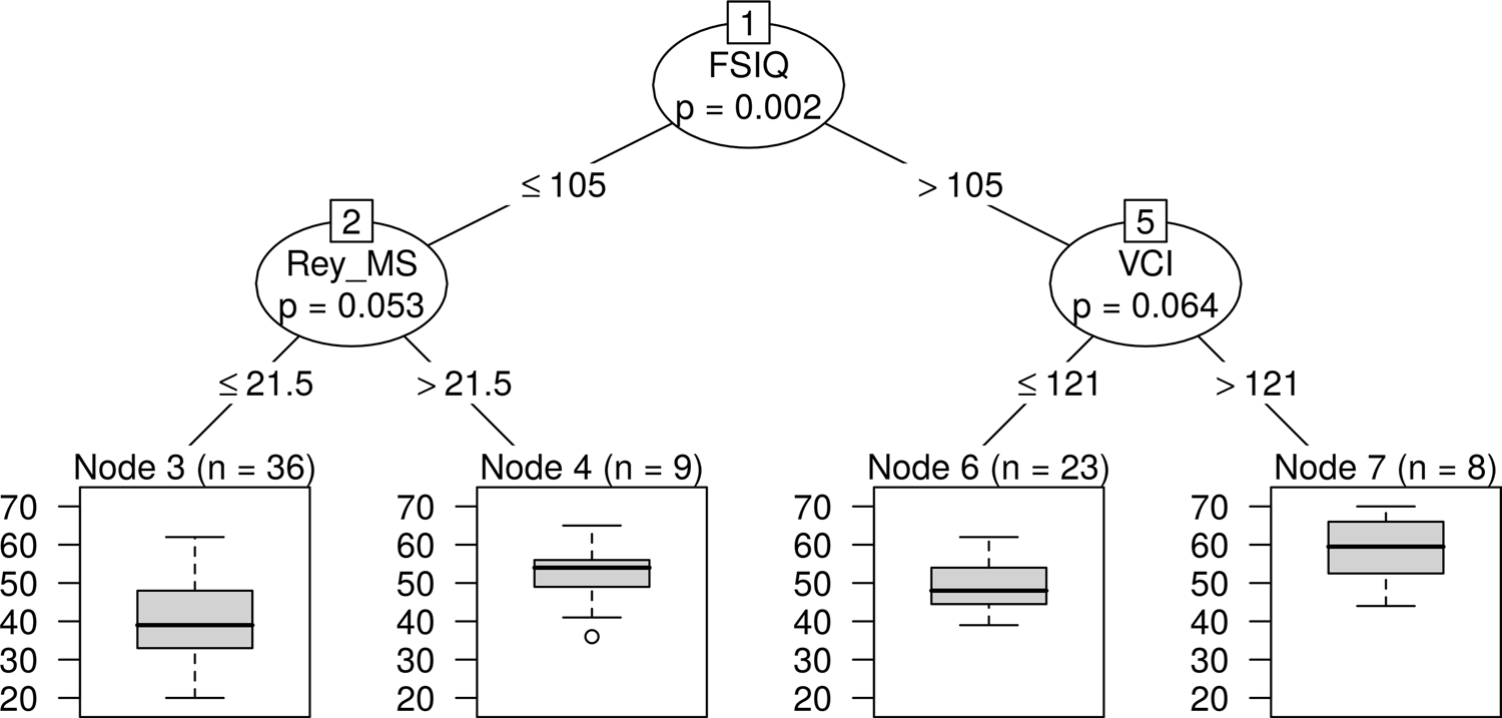
CART decision tree for spelling accuracy

For spelling accuracy RF identified by FSIQ, Rey MS, and VCI as explanatory variables. According to the CART IQs > 105 provide suitable bases for accurate spelling. The role of the FSIQ is moderated by the recall accuracy of the Rey figures and the working memory (VCI). The ideal background structure for spelling accuracy is Rey MS, if FSIQ > 105 and VCI > 121, or if FSIQ < 105 and Rey MS > 21.5 (Fig. 3).

### Spelling speed

**Fig. 4:**
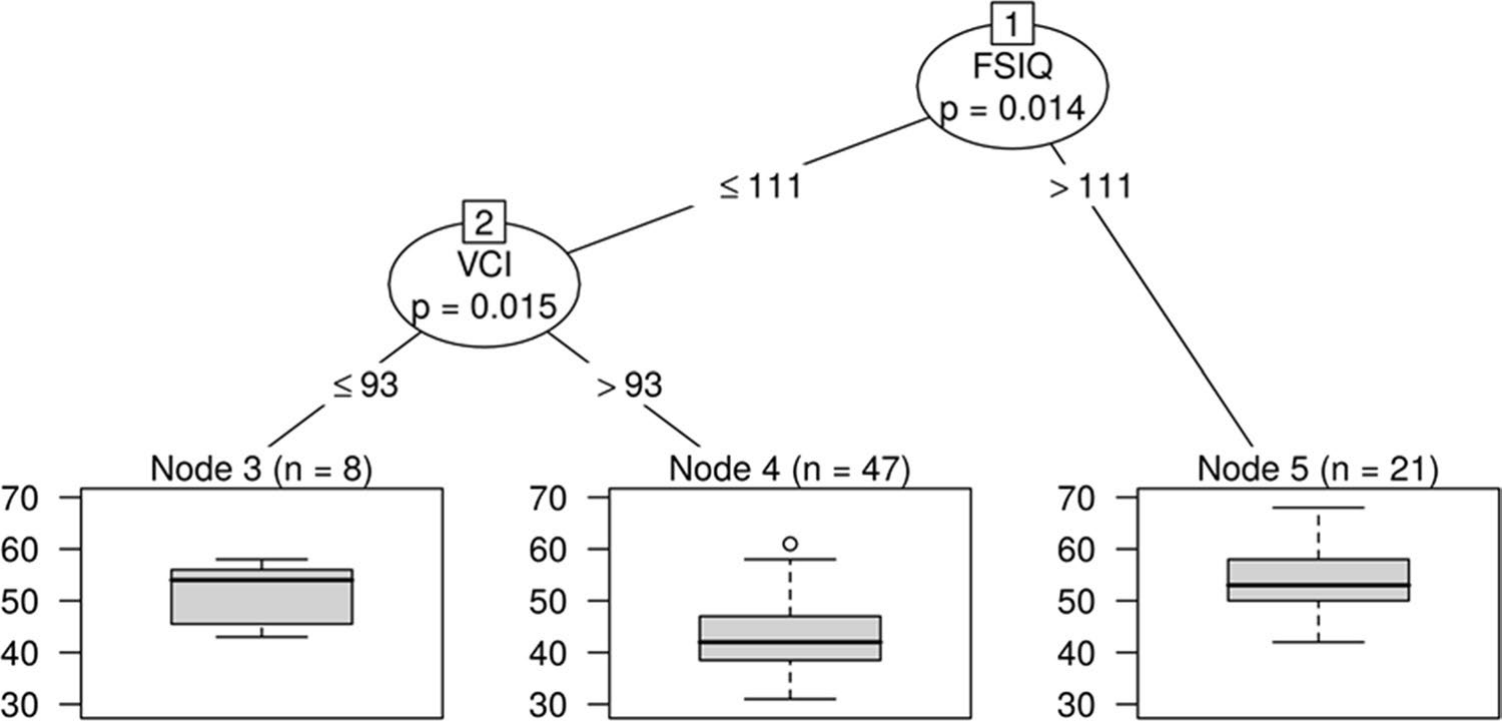
CART decision tree for spelling speed

At the first step for spelling speed from among the heavily intercorrelated independent variables the PrSI was eliminated by the Random Forest; then, CART selected FSIQ and VCI as significant predictors. FSIQ was the main explanatory variable and VMI was a mediator: In order to react quickly, children either needed high FSIQ (> 111) or at least relatively high VCI (> 93) (Fig. 4).

As it was noted earlier, the aim of the study did not require the groups to be matched on IQ.

Nevertheless, we checked a potential bias in the data resulted by the higher IQs in the good reader group and lower IQs in the other two groups. We made subsets from the groups by filtering out children with high and low IQs. According to the repeated analysis using this restricted data set (preterms *n* = 13, dyslexics *n* = 17, good readers *n* = 20), the mean values hardly changed after the removal of children with high and low IQs. The results of the comparisons of the cognitive measures in the three groups, using the same general linear mixed models as in case of the total sample, were as follows: No significant difference was found in FSIQ (*p* = 0.8454), VCI (*p* = 0.4849), PRI (*p* = 0.2673), WMI (*p* = 0.0954), PSI (*p* = 0.3002), Rey CT (*p* = 0.0675), Rey_MT (*p* = 0.0846). However, the differences remained significant in reading accuracy (*p* < 0.0001), reading fluency (*p* < 0.0001), spelling accuracy (*p* = 0.0066), and spelling speed (*p* = 0.0042), as well as in Rey CS (*p* = 0.0023) and Rey MS (*p* = 0.046). These results suggested that the group differences in IQ were very unlikely to influence the results of the analyses (which would not have been feasible with the small data set).

## Discussion

None of the groups had any serious cognitive deficits, but the mean performances of the three groups, even though all within the normal range, had the same order in each of the cognitive measures (Table 1). The good readers were the best, followed by the preterms, and the dyslexics lagged behind. However, the advantage of the good readers over the preterms did not reach significance in working memory, processing speed, Rey copy time, and Rey memory time, and was only marginally significant in Rey memory score. It is notable though that the mean verbal comprehension index in the preterms came very close to that in the dyslexics.

The findings of the study did not confirm our expectation based on the majority of published research results that VLBW prematurity should necessarily lead to developmental disadvantages in the acquisition of reading and spelling skills (Aarnoudse-Moens et al. 2006; Breslau et al. 2001; Kovachy et al. 2014; Nosarti et al. 2010; Saigal et al. 1991; Samuelsson et al. 2006; Takeuchi et al. 2016; O’Keeffee et al. 2003).

In reading accuracy and fluency, as well as in spelling accuracy and spelling speed, our Hungarian preterm children performed at the same level as their non-risk counterparts (Table 3). Nevertheless, these findings are not unique as they are in line with the results reported by Kesler et al. (2004), Lee et al. (2017), and Taylor et al. (2011). In trying to find an explanation for the lack of any deficit in the reading and spelling performances of the preterm children it should be noted that our preterm sample was not born at very high risk despite the very low birthweights, and it did not include individuals with neurological impairments or mental retardation. However, it may not be a convincing argument as in the meta-analysis by Kovachy et al. (2014), and the exclusion of children with intellectual impairments did not erase the significant advantage of the full-term children over the preterms. Nevertheless, it should be kept in mind that the samples of the different studies are never directly comparable (Table 4).

**Table 3:** Mean scores and (SDs) of reading and spelling

Group (*n*)	Reading accuracy	Reading fluency	Spelling accuracy	Spelling speed
Preterm (23)	52.6 (6.96)	53.3 (6.13)	51.5 (8.54)	49.2 (7.58)
Dyslexic (27)	31.1 (8.26)	33.5 (7.89)	36.8 (7.15)	41.8 (7.6)
Good readers (30)	54.8 (5.34)	55.5 (5.67)	52 (8.65)	50.7 (8.23)

**Table 4:** Results of the GLMM’s ANOVA tests for the reading and spelling measures

Measure	numDF	denDF	*F*	*p*
Reading accuracy	2	70	80.05	< 0.0001
Reading fluency	2	69	89.24	< 0.0001
Spelling accuracy	2	66	28.93	< 0.0001
Spelling speed	2	66	8.35	< 0.0001

It seems to be more feasible to spot the cognitive mechanisms which contributed to the good performances of the preterms in the reading and spelling tasks. The 3-step analysis including the Random Forest, the Classification and Regression Tree (CART), and the general linear mixed model proved to be fruitful for that purpose.

The cognitive assets of the preterm children were likely to be their rather good spatial–visual memory, working memory, and processing speed which appeared to be the cognitive factors underlying the reading abilities. The lack of any drawback of the preterm children in spelling is more difficult to interpret since the most powerful predictor of both spelling accuracy and spelling speed was the WISC-IV full-scale IQ in which the preterms as a group scored significantly lower. The background of spelling seems to be more complex as compared to reading as the full-scale IQ is a very complex measure in itself. The relatively better spatial–visual memory which acted as a mediator may have helped the preterms with lower IQs to acquire more accurate spelling. In spelling speed the key of the good performances of the preterms is probably the working memory. The analyses showed that children with lower IQs had a chance for good spelling speed if they had higher scores in working memory, and in the latter measure the preterm group did not lag behind the non-risk good readers.

The inclusion of diagnosed dyslexics in a study aiming at the understanding of the development of reading and spelling skills in preterm children—which, to our knowledge, was an unprecedented idea—proved useful. The difficulties of the dyslexic children in reading and spelling are readily explained by their cognitive handicaps. They scored significantly lower as compared to the good readers in each of the cognitive measures identified by the multivariate analysis as underlying the reading and spelling competencies. The performances of the dyslexics corroborated the power of the prediction models. The use of the Random Forest, the CART technique, and the linear mixed model represented a novel approach in the research aiming to reveal the cognitive mechanisms underlying reading and spelling skills.

Understanding the cognitive background of the good reading and spelling performances is of crucial importance for designing intervention for children with reading and spelling deficits.

As far as the chances of preterm children to acquire good reading and spelling skills are concerned, we can only speculate about. We had no reliable information about our subjects’ history of participating in intervention programs, but as they are widely available in Hungary and it is quite likely that these preterm children, who were regularly checked as subjects of a follow-up project, were referred to early intervention if there was any indication of need for it. A meta-analysis demonstrated the beneficial impacts of early intervention on cognitive outcomes, which lasted until school age (Spittle et al. 2015). The neurophysiological basis of the effectiveness of early intervention is neuronal plasticity which is enhanced in the developing brain. Overproduction of synapses in infancy and childhood leads to increased plasticity by providing an excess of synapses until early adolescence (Johnston 2009). The survival of new neurones is supported by environmental stimulation (Vaccarino and Ment 2004). Apart from organized interventions parents can also provide an enriched environment if they are aware of their preterm children’s special needs (Finch-Edmondson et al. 2019; Kalmár 1996).

Because of the small sample size, the generalizability of our reassuring results on the reading and spelling skills in the VLBW preterm children is limited. Another limitation of the study is that our measurements did not go beyond the single-word processing stage of reading and spelling acquisition, and it has to be kept in mind that in risk children the development often takes unexpected turns and after a period of problem-free growth signs of the risk status may return (“sleeper effect,” Wrape 2003, “moving risk,” Gordon and Jens 1988). Therefore, the long-term follow-up of all children born at perinatal risks is warranted.
